# Characterization of a prenatally assessed de novo supernumerary minute ring chromosome 20 in a phenotypically normal male

**DOI:** 10.1186/1755-8166-2-1

**Published:** 2009-01-07

**Authors:** Sofia Kitsiou-Tzeli, Emmanouil Manolakos, Magdalini Lagou, Maria Kontodiou, Nadezda Kosyakova, Elisabeth Ewers, Anja Weise, Antonios Garas, Sandro Orru, Thomas Liehr, Aikaterini Metaxotou

**Affiliations:** 1 Department of Medical Genetics, University of Athens, Aghia Sofia Children's Hospital, GR-11527, Athens, Greece; 2 Bioiatriki S.A, Kifisias Av. 132 and Papada, GR-115 26 Athens, Greece; 3 Institute of Human Genetics and Anthropology, Kollegiengasse 10, D-07743 Jena, Germany; 4 Department of Obstetrics and Gynecology, University of Thessaly, Larissa, Greece; 5 Department of Medical Genetics, University of Cagliari, Binaghi Hospital, Cagliari, Italy

## Abstract

**Background:**

The heterogeneous group of small supernumerary marker chromosomes (sSMCs) presents serious counseling problems, especially if they are present de novo and diagnosed prenatally. The incidence has been estimated at 1 in 1000 prenatal samples. We present a case of mosaic sSMC diagnosed prenatally after amniocentesis. The sSMC was characterized by various molecular cytogenetic techniques and determined to be a r(20) chromosome. After genetic counseling, the parents decided to continue the pregnancy, and a boy with minor phenotypic variants was born after 39 weeks of pregnancy. The case is compared with four other cases of prenatally detected r(20) mosaicism.

**Results:**

Here we describe a 3 months old male child with normal pre- and postnatal development and with a de novo ring supernumerary marker chromosome in amniocytes cultures. Using new fluorescence in situ hybridization (FISH) techniques, three distinguishable sSMCs (cryptic mosaicism), all derived from chromosome 20, were observed, including ring and minute chromosomes. This heterogeneity was impossible to detect by the conventional G-banding technique or conventional FISH technique that were used before the application of new FISH techniques (subcentromere-specific multicolor-FISH [subcenM-FISH]) and a probe, specific for the 20p12.2 band. The sSMC present in 25% of the cells was present as r(20)(::p12.2~12.3->q11.1::)[5]/r(20;20)(::p12.1->q11.1::q11.1 >p12.1::)[2]/min(20;20)(:p12.1->q11.1::q11.1->p12.1:)[1].  The final karyotype was 47,XY,+r(20)[25%]/46,XY[75%].

**Conclusion:**

We emphasize the importance of application of molecular cytogenetics in a prenatally diagnostic laboratory and description of more cases to enable a better genetic counseling and risk evaluation.

## Background

Small supernumerary marker chromosomes (sSMCs) are structurally abnormal chromosomes that cannot be identified or characterized unambiguously by conventional cytogenetics alone, and they are generally equal in size or smaller than chromosome 20 at the same metaphase spread [1]. The heterogeneous group of sSMCs presents serious genetic counseling problems, especially if they are present de novo and diagnosed prenatally. The incidence of sSMCs has been estimated at 0.075% in prenatal diagnoses [2]. Identification of an sSMC only by cytogenetics is almost impossible. For this reason fluorescence in situ hybridization (FISH) is most valuable and has been successfully applied for the determination of the chromosomal origin of sSMCs [3].

Most marker chromosomes are derived from the short arms and pericentric regions of the acrocentric chromosomes, while the occurrence of an additional derivative chromosome 20 is rare. No common phenotype of sSMC(20) has been established [3,4]. So far there are only four reports of an extra r(20) ascertained prenatally [5-7].

Here we describe a 3 months old infant who had a mosaic karyotype detected prenatally 47,XY,+r(20)/46,XY. The results of the clinical, molecular cytogenetic and molecular findings are presented and compared to reports previously published.

## Case presentation

The proposita, a 36-year-old woman was referred for amniocentesis at 16 weeks of gestation because of advanced maternal age. A previous pregnancy resulted in birth of a healthy daughter. The woman and her 43-year-old husband were healthy, non-consanguineous and had no family history of genetic disorders and congenital malformations. Detailed ultrasonography, as well as fetal echocardiography at 21 weeks of gestation, showed a normally developed fetus with no obvious morphologic abnormalities. After genetic counseling, the family decided to continue the pregnancy to term. A boy was born after an uneventful 39-weeks gestation with a birth weight of 3450 g (50^th ^centile), length 51 cm (50^th ^centile), head circumference 34 cm (50^th ^centile) and Apgar score 9 at 1^st ^minute.

Evaluation shortly after birth revealed lack of dysmorphic facial features, with simian crease and unilateral metatarsus varus as the only phenotypic variants. No abnormalities were noted at the brain ultrasonography and evoked acoustic potentials. After 3 months his growth was normal (weight 6290 g, length 61.5 cm, head circumference 40 cm). He was able to maintain head control, follow moving objects, recognize his parents, and react positively to stimuli.

## Results

**Figure 1:**
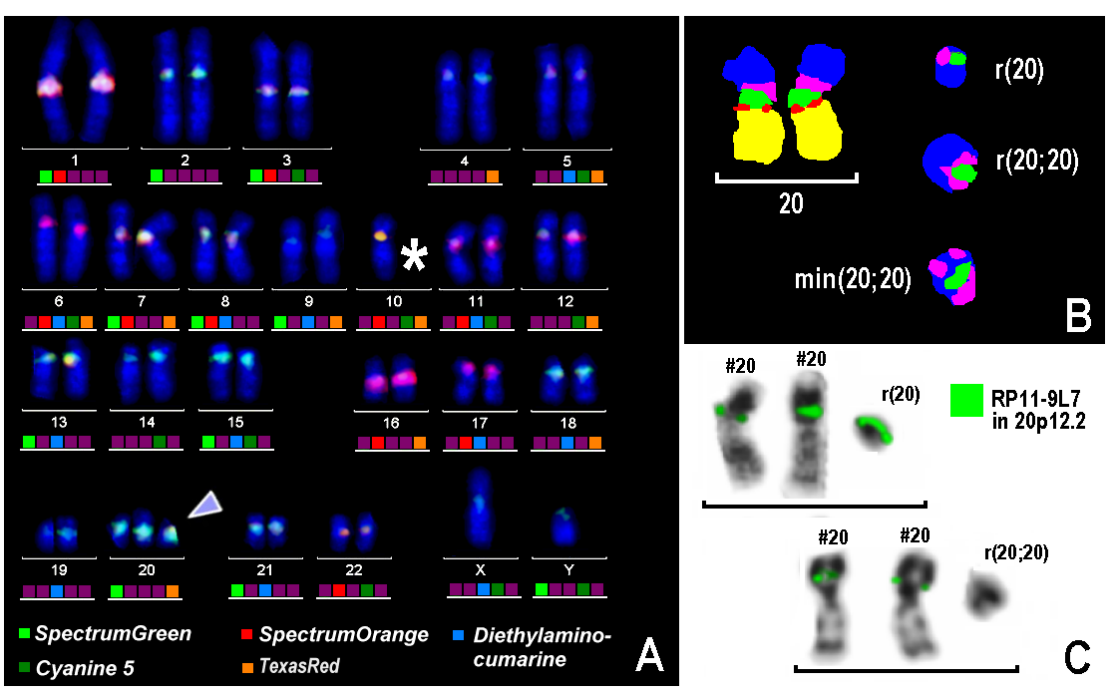
**A) cenM-FISH identified the sSMC as a derivative of chromosome 20 (arrowhead)**. The asterisk besides chromosome 10 indicate that one chromosome is lacking here due to preparation artifacts. B) subcenM-FISH with a chromosome 20 specific probe set revealed the presence of three different types of marker chromosomes besides two normal chromosomes 20, i.e. either a ring (r(20)), a double ring (r(20;20)) or an inverted duplicated dicentric minute chromosome 20 (min(20;20)). C) A BAC probe specific for 20p12.2 showed a signal only in the r(20) but not on r(20;20) or min(20;20). Thus, a final karyotype of r(20)(::p12.2~12.3->q11.1::)[5]/r(20;20)(::p12.1->q11.1::q11.1->p12.1::)[2]/min(20;20)(:p12.1->q11.1::q11.1->p12.1:)[1] for the marker chromosome was defined.

Cytogenetic analysis of amniotic cells revealed two cell lines. The karyotype was 46,XY in 75% of the analyzed mitoses, while an additional monocentric chromosome (marker) was noted in 25% of the cells, karyotype 47,XY,+mar [25%]/46,XY [75%]. The parental karyotypes were normal (blood lymphocytes). Also non-paternity was excluded (see below). Thus, the marker probably arose de novo. To identify the origin of the de novo sSMC, FISH analysis was performed. Fluorescence in situ hybridization studies using centromere-specific multicolor FISH were applied [8] (Fig. 1). The chromosome 20 origin was confirmed by application of a commercially available probe for the centromeric region of chromosome 20. The shape and size of the sSMC were further delineated using a probe set containing centromere-near probes in a subcentromere-specific probe set [9], the BAC probe RP11-9L7 in 20p12.2 and subtelomeric probes for 20pter and 20qter. All commercially available probes were obtained from Abbott/Vysis. So, overall the sSMC present in 25% of the cells was present as a cryptic mosaic (three distinguishable sSMCs, non detectable by the conventional G-banding technique or conventional FISH technique, all derived from chromosome 20) as r(20)(::p12.2~12.3->q11.1::)[5]/r(20;20)(::p12.1->q11.1::q11.1->p12.1::)[2]/min(20;20)(:p12.1->q11.1::q11.1->p12.1:)[1].

## Discussion

Since the first report of an sSMC(20) by Callen et al [10], overall 42 more cases (including the present case) have been described, most of them detected postnatally [11]. While 14 cases were clinically normal, 24 cases were abnormal (including one neocentric sSMC(20)) and 4 had an unclear clinical correlation. The most common findings in the abnormal cases were growth retardation and delayed psychomotor development.

Karyotype/phenotype correlations have been extremely difficult to establish in sSMC cases in general, due to their infrequency [12]. The two patients reported by Viersbach et al [6] with r(20) mosaicism prenatally detected, were phenotypically and developmentally normal at the age of 20 months, Table 1, cases 2, 3. The first patient had a ring chromosome, containing a small amount of euchromatic material, and the second patient was carrier of a small, metacentric and most probably heterochromatic marker. Similar was the case reported by Cotter et al [7] with a karyotype of 47,XY,+mar[3]/46,XY[17] reported to be normal at birth, Table 1, case 5. The fourth case of a prenatally detected 46,XY/47,XY,+r(20)/47,XY,+20/48,XY,+2r(20), showed at the age of 16 months delayed psychomotor development, physical anomalies and growth retardation [5] Table 1, case 4.

**Table 1:** Cytogenetic finding and clinical data in 5 prenatal cases with a supernumerary der(20).

**Case**	**Age**	**Karyotype**	**Cell system Mosaic finding in %**	**Clinical finding**	**Reference**
1	8 months	46,XY/47,XY, +r(20)(::p12.2~12.3->q11.::)/ 47,XY, +r(20)(::p12.1-> q11.1::q11.1->p12.1.::)/ 47,XY,+min(20)(:p12.1-> q11.1::q11.1->p12.1:) de novo	Amnyocytes (75:25%)	Normal psychomotor development	Present investigation

2	20 months	46,XY/47,XY,+r(20 de novo	Amniocytes (20:80%) Cord bllod (91:9%)	Normal psychomotor development	Viersbach et al 1997

3	20 months	46,XY/47,XY,+der(20 de novo	Amniocytes (20:80%)	Normal psychomotor development	Viersbach et al 1997

4	16 months	46,XY,/47,XY, +r(20)/47,XY,+20/48,XY,+2r(20)	Amniocytes (10,5:44,7:44,7:0%) Chorionic villi (5,5:16,5:78,0:0%) Amnion (0:36,0:64,0:0%) Skin (16,7:80,6:1,6:1,1%) Cord blood (13,7:86,3:0:0%)	Delayed psychomotor development, height and below 3^rd ^centile, hypotonia, asymmectric triangular face, prominent forehead, bulbous nose with slightly upturned tip, hypoplastic short philtrum, small mouth, high palate, micro- and retrognathia, abnormal ears, proximally placed adducted thumbs, clinodactyly lymphedema on the dorsa of feet, abnormal position of toes, prominent heels, increased skin elasticity, hyperextensible joints	Batista et al 1995

5	6 months	46,XY/47,XY,+der(20) de novo	Amniocytes (75:15%)	Normal psychomotor development	Cotter et al 2005

Our patient had normal pre- and postnatal development and does not present any of the phenotypic features of the described cases of mosaic extra ring 20 chromosome, nor psychomotor delay [12]. However, further developmental follow-up is warranted.

The degree of mosaicism is a critical element in the determination of phenotype in sSMC cases [13]. In addition, it is generally accepted that it is the presence of euchromatin that makes a marker chromosome deleterious to the phenotype [14]. However, there is data on centromere-near and other regions being harmless if present in additional copies [4,15]. Although the clinical consequences of small markers containing regions adjacent to the centromere are not clear, the r(20) of our case was composed mainly of heterochromatic centromere region, and therefore is expected to be benign.

Limited data currently do not permit consistent genotype-phenotype correlations to be made [11]. The identification of more cases with der(20) chromosomes is needed for further interpretation and may allow useful phenotypic comparisons to be made. Molecular cytogenetics in combination with other molecular studies can provide valuable information about the chromosomal origin and the composition of sSMCs. Liehr [16] states the importance of application of molecular cytogenetics in a prenatally diagnostic laboratory and suggests a straightforward scheme to characterize at least the chromosomal origin as quickly as possible and to compare the actual case with similar cases from the literature. As more sSMCs are classified and more data are collected, better genetic counseling and risk evaluation can be offered.

## Methods

### Cytogenetic and fish studies

Cytogenetic study was carried out on amniocytes by G-banding according to standard procedures, and 100 G-banded metaphases were analyzed. To identify the origin of the de novo sSMC, FISH analysis was performed. The chromosomal origin was determined using centromere-specific multicolor FISH [8]. The chromosome 20 origin was confirmed by application of a commercially available probe for the centromeric region of chromosome 20. The shape and size of the sSMC were further delineated using a probe set containing centromere-near probes in a subcentromere-specific probe set [9], the BAC probe RP11-9L7 in 20p12.2 and subtelomeric probes for 20pter and 20qter. All commercially available probes were obtained from Abbott/Vysis.

### Molecular studies

Parental blood samples were collected and genomic DNA was extracted using the NucleoSpin blood extraction kit (Macherey-Nagel GmbH & Co. KG, Düren, Germany). DNA from amniocytes was isolated using an InstaGene Matrix kit (Bio-Rad Laboratories, Hercules, CA, USA). Uniparental disomy (UPD) of the normal chromosomes 20 was excluded by means of parent-to-fetus segregation analysis using a panel of 8 polymorphic markers located along the length of chromosome 20 (D20S103, D20S117, D20S199, D20S194, D20S195, D20S109, D20S193, D20S200). Quantitative fluorescence (QF) PCR was performed to amplify the repeat sequences at the above polymorphic loci and the primer sequences were probed with fluorescent labels as described elsewhere [17].

## Consent

This case report is presented with the consent of the patient's family.

## Competing interests

The authors declare that they have no competing interests.

## Authors' contributions

SKT conceived of the study and participated in its design and coordination. EM performed the cytogenetic studies and participated in its design and coordination. ML and MK participated in the cytogenetic analysis. NK, EE and AW participated in the molecular cytogenetic analysis. AG performed the ultrasonography of the pregnant woman. SO performed the molecular analysis for uniparental disomy. TL was responsible for the molecular cytogenetic studies and participated in the drafting of the manuscript. AM participated in the design and coordination of the study and participated in the drafting of the manuscript. All authors read and approved the final manuscript.
