# Correction: Characterization of a prenatally assessed de novo supernumerary minute ring chromosome 20 in a phenotypically normal male

**DOI:** 10.1186/1755-8166-2-8

**Published:** 2009-02-20

**Authors:** Sofia Kitsiou-Tzeli, Emmanouil Manolakos, Magdalini Lagou, Katerina Anagnostopoulou, Maria Kontodiou, Nadezda Kosyakova, Elisabeth Ewers, Anja Weise, Antonios Garas, Sandro Orru, Thomas Liehr, Aikaterini Metaxotou

**Affiliations:** 1 Department of Medical Genetics, University of Athens, Aghia Sofia Children's Hospital, GR-11527, Athens, Greece; 2 Bioiatriki S.A, Kifisias Av. 132 and Papada, GR-115 26 Athens, Greece; 3 Genomedica S.A, Kanari, 5-7, 18537, Piraeus, Greece; 4 Institute of Human Genetics and Anthropology, Kollegiengasse 10, D-07743 Jena, Germany; 5 Department of Obstetrics and Gynecology, University of Thessaly, Larissa, Greece; 6 Department of Medical Genetics, University of Cagliari, Binaghi Hospital, Cagliari, Italy

## Correction

After publication of this work [1], we noted that we inadvertently failed to include the complete list of all coauthors. The full list of all authors has now been added and the Authors' contributions and Competing interests section modified accordingly.

## Competing interests

The authors declare that they have no competing interests.

## Authors' contributions

SKT conceived of the study and participated in its design and coordination. EM performed the cytogenetic studies and participated in its design and coordination. ML and MK participated in the cytogenetic analysis. NK, EE and AW participated in the molecular cytogenetic analysis. AG performed the ultrasonography of the pregnant woman. SO and KA performed the molecular analysis for uniparental disomy. TL was responsible for the molecular cytogenetic studies and participated in the drafting of the manuscript. AM participated in the design and coordination of the study and participated in the drafting of the manuscript. All authors read and approved the final manuscript.
